# Thyroid V_50_ is a risk factor for hypothyroidism in patients with nasopharyngeal carcinoma treated with intensity-modulated radiation therapy: a retrospective study

**DOI:** 10.1186/s13014-020-01490-x

**Published:** 2020-03-23

**Authors:** Ling Zhou, Jia Chen, Wei Shen, Zheng-Lu Chen, Shuang Huang, Chang-Juan Tao, Ming Chen, Zhong-Hua Yu, Yuan-Yuan Chen

**Affiliations:** 1 Institute of Cancer and Basic Medicine (ICBM), Chinese Academy of Sciences, Hangzhou, China; 2 Department of Radiation Oncology, Cancer Hospital of University of Chinese Academy of Sciences, Hangzhou, China; 3 Department of Radiation Oncology, Zhejiang Cancer Hospital, Hangzhou, China; 4 Postgraduate Education, Guangdong Medical University, Zhanjiang, China; 5 Hangzhou YITU Healthcare Technology Co, Ltd, Hangzhou, China; 6 Department of Oncology, the Affiliated Hospital of Guangdong Medical University, Zhanjiang, China

**Keywords:** Nasopharyngeal carcinoma, Hypothyroidism, Radiation therapy

## Abstract

**Background:**

We investigated the risk factors of radiation-induced thyroid dysfunction, then combined the clinical factors and optimum thyroid dosimetric parameters to predict the incidence rate of hypothyroidism (HT) and to guide individualized treatment.

**Methods:**

A total of 206 patients with histologically proven nasopharyngeal carcinoma (NPC) treated at the Cancer Hospital of the University of Chinese Academy of Sciences between January 2015 and August 2018 were included. Dose–volume histogram (DVH) data, including mean dose, absolute volume, V_20_, V_25_, V_30_, V_35_, V_40_, V_45_, V_50,_ V_55_, and V_60_ were extracted and used as dosimetric parameters. A logistic regression analysis model was built to identify predictors related to HT occurring within 2 years.

**Results:**

Sex, N stage, thyroid volume, mean thyroid dose, and thyroid V_20_ and V_50_ were significantly different between patients with and without HT. Logistic regression analysis showed that N stage, thyroid volume, and thyroid V_50_ were independent predictors of HT. The radiosensitivity of the thyroid decreased as the thyroid volume increased. Patients with N stage > 1 had significantly higher HT incidence (37.38%) than patients with N stage ≤1 (13.11%). The incidence of HT was 54.55% in patients with thyroid V_50_ > 24% and was 34.15% in patients with thyroid V_50_ ≤ 24%.

**Conclusions:**

The incidence of HT is significantly associated with N stage, thyroid volume, and thyroid V_50_. More attention should be paid to patients with NPC with thyroid volume ≤ 12.82 cm^3^ and advanced N stage disease.

## Background

Nasopharyngeal carcinoma (NPC) is common in southern China. Radiotherapy is the primary treatment, and the 5-year overall survival rate is up to about 80% [1, 2]. Radiation inevitably damages normal tissue while treating tumors. Radiation-induced thyroid abnormality is a common complication in patients with NPC who have undergone radiotherapy, and has been reported since 1929 [3]. In terms of the quality of life (QoL) of patients with NPC who have receive radiation, many clinical studies have shown that intensity-modulated radiotherapy (IMRT) can preserve many organs at risk (OARs) better than conventional radiotherapy and improve the patients’ QoL [4–6]. However, it is not easy to protect the pituitary and thyroid glands, as they are close to the primary tumor and neck nodal metastasis. However, it is worth noting that IMRT may result in a higher incidence of subclinical hypothyroidism (HT) than three-dimensional conformal radiotherapy (51.1% vs 27.3%) [7].

The incidence of HT in patients with NPC treated with IMRT ranges from 14.1 to 60% [8, 9], mostly occurring within 5 years after treatment. The incidence is high 1–2 years after radiotherapy [7, 10, 11]. The most common symptoms of HT are fatigue, lethargy, cold intolerance, weight gain, constipation, change in voice, and dry skin [12]. Serious HT may increase the risk of heart disease and influence the progression-free survival and overall survival of patients with cancer [11, 13]. Many studies have focused on the factors affecting thyroid function; nevertheless, the impact of radiation on HT related to the thyroid and pituitary glands remains poorly understood, and the incidence of HT has not been controlled effectively in patients with NPC post-IMRT [14]. The purpose of the present study was to investigate the risk factors related to radiation-induced thyroid dysfunction, then combine the clinical factors and optimum thyroid dosimetric parameters to predict the incidence of HT and guide individualized treatment.

## Methods and materials

### Patient selection

We collected the information of patients with histologically proven NPC from the Cancer Hospital of the University of Chinese Academy of Sciences between January 2015 and August 2018. We included patients with complete clinical information (including clinical characteristics, pre-radiotherapy biochemical results of normal thyroid function test), pathologically confirmed primary NPC, and follow-up for > 1 year or who developed HT within 1 year. Patients who had undergone chemotherapy or radiotherapy before treatment at our hospital or who had problems with the hypothalamic-pituitary-thyroid (HPT) axis were excluded. In total, 206 patients were included in this analysis.

### Treatment

#### Chemotherapy

All patients received 2–3 cycles of TP (docetaxel/paclitaxel + cisplatin/nedaplatin), PF (cisplatin/nedaplatin + fluorouracil), or TPF (docetaxel/paclitaxel + cisplatin/nedaplatin + fluorouracil) induction chemotherapy, followed by IMRT with concurrent cisplatin or nedaplatin chemotherapy.

#### Radiotherapy

All patients received radical IMRT. The patients were immobilized in the supine position with a head-neck-shoulder thermoplastic mask; computed tomography simulation (CT-sim) scan from the top of the head to the sternal angle was performed using a Philips Brilliance CT with 3-mm thickness; magnetic resonance imaging (MRI) scan was obtained in the same posture and immobilization mode with a Siemens Verio 3.0 T. The CT-sim and MRI scan images were transmitted into RayStation 4.0. PGTVnx+rn (planning gross primary tumor and retropharyngeal lymph node volume), PGTVnd (planning positive lymph nodes), PTV-1 (planning target volume 1), PTV-2, and OARs were contoured based on CT. According to RTOG (Radiation Therapy Oncology Group) 0615 [15], the PGTVnx+rn included the primary nasopharyngeal tumor and retropharyngeal lymph nodes with a prescription dose of 70.40 Gy at 2.20 Gy per fraction, the PGTVnd included positive lymph nodes with a prescription dose of 68.80 Gy at 2.15 Gy per fraction, the PTV-1 were high-risk areas included the primary nasopharyngeal tumor and nasopharyngeal mucosa with a prescription dose of 64.00 Gy at 2.00 Gy per fraction, the PTV-2 were low-risk areas with a prescription dose of 54.40 Gy at 1.70 Gy per fraction, included posterior maxillary sinus, pterygopalatine fossa, parapharyngeal space, skull base, part of posterior ethmoid sinus, bilateral lymphatic drainage area and so on. All patients were treated with daily fractions over 5 days per week using a linear accelerator (6–8 MV). The OAR dose constraints were as follows: the maximum temporal lobe dose was < 60 Gy or D1cc (the dose delivered volume of 1 cm^3^, Gy) was < 65 Gy, and the maximum pituitary dose was < 50 Gy; the dose constraints of other OARs were based on expert consensus, and there has been no thyroid dose constraint in clinical practice to date.

### Dosimetric parameters

A senior doctor re-contoured the pituitary and thyroid. Dose–volume histogram (DVH) data, including the mean dose, absolute volume, V_20_, V_25_, V_30_, V_35_, V_40_, V_45_, V_50_, V_55_, and V_60_ (V_x_: percentage volume of organ receiving more than x Gy,) were extracted and used in the statistical analysis.

### Thyroid function test

Thyroid function was assessed by monitoring serum thyroid-stimulating hormone (TSH), free triiodothyronine (FT_3_), and free thyroxine (FT_4_). Hypothyroidism was defined as having TSH concentrations above the reference range (0.380~4.340 μIU/mL) and FT_4_ concentrations within or lower than the normal range (0.81–1.89 ng/dL). The above definitions cover both clinical and subclinical HT [12].

### Statistical analysis

First, clinical characteristics stratified by HT were described via means and standard deviations (for normally distributed variables), interquartile range [median (Q25–Q75)] (for abnormally distributed variables), or frequency and percentages (for categorical variables), and their differences were compared by *t*-test, Mann-Whitney U test, or chi-square (or Fisher’s exact probability) test, respectively. Second, a logistic regression model was performed to examine which clinical and dosimetric parameters were related to the development of HT. Only characteristics significantly different in univariate analysis were included in the logistic regression model, and the independent predictors of HT were identified using backward elimination (*P* > 0.1 was excluded). Third, the dosimetric parameter cut-offs were calculated using the receiver operating characteristics (ROC) curve. Finally, we reclassified the significantly independent variables into binary variables according to the cut-offs, and evaluated their association with HT using the chi-square test (for categorical variables) or Cochran-Armitage trend test (for ordinal variables). *P* < 0.05 was considered statistically significant. All analyses were performed using R-3.6.0.

## Results

### Patients’ characteristics

Table 1 presents the descriptive statistics. Of the total 206 patients with NPC, 135 were male and 71 were female, and the patient age range was 25–82 years. The follow-up time was 6–48 months, with a median of 19 months. Overall, 50.49% of the patients (104/206) developed subclinical HT. The average thyroid and pituitary dose was 45.88 Gy (range, 42.86–47.98 Gy) and 51.91 Gy (range, 40.70–59.24 Gy), respectively. Sex, N stage, volume, average dose, and the thyroid V_20_ and V_50_ were significantly different between patients with and without HT. No differences were shown for other factors such as age, T stage, clinical stage, chemotherapy, and pituitary gland dosimetric parameters among the patients with and without HT.

**Table 1:** Clinical characteristics in patients with NPC treated with IMRT

Variable	Patients		Statistic	*P*-value
	With HT (*N* = 104)	Without HT (*N* = 102)		
Age (mean ± SD)	50.62 ± 10.75	52.21 ± 10.67	*t* = 1.070	0.289
Sex			χ^2^ = 0.017	0.017^*^
Male	60 (29.13)	75 (36.41)		
Female	44 (21.36)	27 (13.11)		
T stage (2010UICC)			χ^2^ = 0.004	0.947
T_1–2_	19 (9.22)	19 (9.22)		
T_3–4_	85 (41.26)	83 (40.29)		
N stage (2010UICC)			χ^2^ = 5.531	0.021^*^
N_0–1_	27 (13.11)	42 (20.39)		
N_2–3_	77 (37.38)	60 (29.13)		
M stage (2010UICC)				0.498^a^
M_0_	98 (47.57)	99 (48.06)		
M_1_	6 (2.91)	3 (1.46)		
Clinical stage (2010UICC)			χ^2^ = 2.158	0.142
I-II	4 (1.94)	9 (4.37)		
III-IV	100 (48.54)	93 (45.15)		
Nimotuzumab			χ^2^ = 0.004	0.947
No	85 (41.26)	83 (40.29)		
Yes	19 (9.22)	19 (9.22)		
Neoadjuvant chemotherapy			χ^2^ = 1.500	0.221
No	4 (1.94)	8 (3.88)		
Yes	100 (48.54)	94 (45.63)		
Thyroid volume (cm^3^)	12.77 (10.79–16.13)	15.88 (13.33–19.97)	*Z* = 4.091	< 0.001^***^
Mean dose of thyroid (Gy)	46.08 (44.68–47.98)	45.67 (42.86–47.05)	*Z* = -1.981	0.048^*^
V_20_	1.00 (1.00–1.00)	1.00 (1.00–1.00)	*Z* = -2.182	0.029^*^
V_25_	1.00 (1.00–1.00)	1.00 (1.00–1.00)	*Z* = -0.798	0.425
V_30_	1.00 (0.97–1.00)	0.99 (0.95–1.00)	*Z* = -1.697	0.090
V_35_	0.93 (0.87–0.98)	0.92 (0.78–0.96)	*Z* = -1.797	0.072
V_40_	0.77 (0.69–0.85)	0.76 (0.59–0.82)	*Z* = -1.808	0.071
V_45_	0.56 (0.49–0.64)	0.54 (0.40–0.60)	*Z* = -1.950	0.051
V_50_	0.33 (0.28–0.41)	0.32 (0.23–0.38)	*Z* = -2.090	0.037^*^
V_55_	0.11 (0.05–0.18)	0.08 (0.05–0.15)	*Z* = -1.344	0.179
V_60_	0.01 (0.00–0.04)	0.00 (0.00–0.02)	*Z* = -1.322	0.186
Pituitary volume (cm^3^)	0.47 (0.36–0.55)	0.43 (0.35–0.53)	*Z* = -1.259	0.208
Mean dose of pituitary (Gy)	51.04 (43.26–57.09)	52.78 (40.70–59.24)	*Z* = 0.770	0.441
V_20_	1.00 (1.00–1.00)	1.00 (1.00–1.00)	*Z* = 0.439	0.661
V_25_	1.00 (1.00–1.00)	1.00 (1.00–1.00)	*Z* = -0.633	0.527
V_30_	1.00 (1.00–1.00)	1.00 (1.00–1.00)	*Z* = -0.420	0.674
V_35_	1.00 (0.88–1.00)	1.00 (0.73–1.00)	*Z* = -0.892	0.372
V_40_	1.00 (0.62–1.00)	1.00 (0.5–1.00)	*Z* = -0.801	0.423
V_45_	0.98 (0.41–1.00)	1.00 (0.43–1.00)	*Z* = 0.501	0.616
V_50_	0.52 (0.00–1.00)	0.81 (0.00–1.00)	*Z* = 0.867	0.386
V_55_	0.10 (0.00–0.56)	0.39 (0.00–0.9)	*Z* = 1.247	0.213
V_60_	0.00 (0.00–0.46)	0.00 (0.00–0.53)	*Z* = 0.823	0.411

^a^*P*-value was calculated using Fisher’s exact probability; *P < 0.05, **P < 0.01, ***P < 0.001

### Independent predictors of HT

The logistic regression analysis revealed that N stage, thyroid volume, and thyroid V_50_ were independent predictors of HT (Table 2). The risk of developing HT was significantly higher in the N_2–3_ cohort than in the N_0–1_ cohort (odds ratio [OR] = 1.91, 95% confidence interval [CI] = 1.02–3.57), and HT was highly prevalent in the thyroid V_50_ > 24% cohort (OR = 8.93, 95% CI = 0.89–89.76) and thyroid volume ≤ 12.82 cm^3^ cohort (OR = 0.89, 95% CI = 0.83–0.94).

**Table 2:** Logistic regression analysis of factors influencing HT in patients with NPC treated with IMRT

Variable	B	SE	*P*-value	OR (95%CI)
N-stage N_2–3_ (vs N_0–1_)	0.65	0.32	0.04^*^	1.91 (1.02–3.57)
Thyroid volume (cm3)	−0.12	0.03	< 0.001^***^	0.89 (0.83–0.94)
Thyroid V_50_	2.19	1.18	0.06	8.93 (0.89–89.76)

*P < 0.05, ***P < 0.001

### ROC analysis

**Fig. 1:**
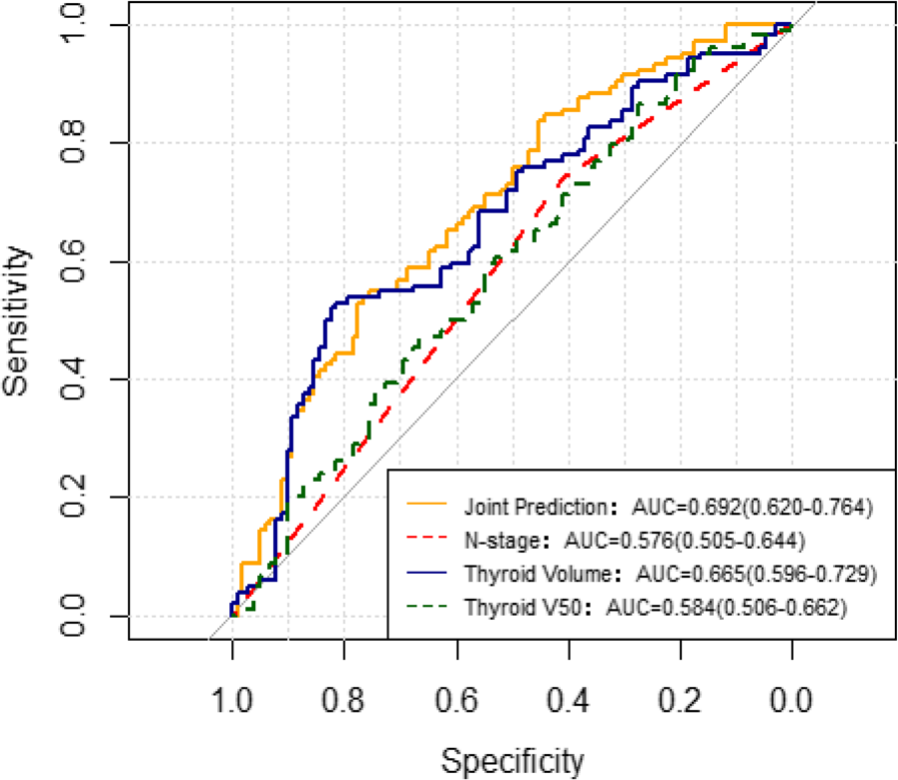
ROC analysis of 206 patients with NPC treated with IMRT

The ROC analysis (Fig. 1) suggested that thyroid volume was a good predictor of HT, with an area under the curve (AUC) of 0.67 (*P* < 0.001). The thyroid volume and thyroid V_50_ cut-offs were 12.82 cm^3^ and 24%, respectively. Thyroid radiosensitivity decreased with increasing thyroid volume, and thyroid volume < 12.82 cm^3^ was a risk factor. The incidence rate of HT was 37.31 and 75% in patients with thyroid volume > 12.82 cm^3^ and thyroid volume ≤ 12.82 cm^3^, respectively. The incidence rate of HT was 54.55 and 34.15% in patients with thyroid V_50_ > 24% and thyroid V_50_ ≤ 24%, respectively (Table 3).

**Table 3:** Associations of thyroid volume and thyroid V_50_ with HT

Variable		HT		χ^2^	*P*-value
		No (%)	Yes (%)		
Thyroid volume	≤12.82 cm3	18	54 (75.00)	26.61	< 0.001^***^
	> 12.82 cm3	84	50 (37.31)		
Thyroid V_50_	≤0.24	27	14 (34.15)	5.47	0.02^*^
	> 0.24	75	90 (54.55)		

*P < 0.05, ***P < 0.001

### Joint prediction for HT

The joint prediction model was based on two variables each, namely thyroid volume and thyroid V_50_, thyroid volume and N stage, and N stage and thyroid V_50_. An increased trend of HT incidence was observed with decreased thyroid volume while thyroid V_50_ increased (*P* < 0.001), and ranged from 29.03% (volume > 12.82 cm^3^ and V_50_ ≤ 24%) to 79.03% (volume ≤ 12.82 cm^3^ and V_50_ > 24%). Similar trends were also found in patients with decreased thyroid volume while N stage increased (*P* < 0.001), ranging from 31.25% (volume > 12.82 cm^3^ and N_0–1_) to 82.35% (volume > 12.82 cm^3^ and N_2–3_). Besides, the HT incidence rate increased as the thyroid V_50_ and N stage increased (*p* < 0.01), ranging from 14.29% (V_50_ ≤ 24% and N_1–2_) to 56.41% (V_50_ > 24% and N_2–3_) (Table 4).

**Table 4:** Joint prediction for HT

Combined factors	HT		Z	*P*-value
	No (%)	Yes (%)		
**Combined thyroid volume and thyroid V**_**50**_				
Volume > 12.82 cm^3^ and V_50_ ≤ 24%	22 (70.97)	9 (29.03)	−5.45	< 0.001^***^
Volume > 12.82 cm^3^ and V_50_ > 24%	62 (60.19)	41 (39.81)		
Volume ≤ 12.82 cm^3^ and V_50_ ≤ 24%	5 (50.00)	5 (50.00)		
Volume ≤ 12.82 cm^3^ and V_50_ > 24%	13 (20.97)	49 (79.03)		
**Combined N stage and thyroid V**_**50**_				
N_0–1_ and V_50_ ≤ 24%	18 (85.71)	3 (14.29)	−2.86	< 0.01^**^
N_0–1_ and V_50_ > 24%	24 (50.00)	24 (50.00)		
N_2–3_ and V_50_ ≤ 24%	9 (45.00)	11 (55.00)		
N_2–3_ and V_50_ > 24%	51 (43.59)	66 (56.41)		
**Combined thyroid volume and N stage**				
Volume > 12.82 cm^3^ and N_0–1_	33 (68.75)	15 (31.25)	−5.50	< 0.001^***^
Volume > 12.82 cm^3^ and N_2–3_	51 (59.30)	35 (40.70)		
Volume ≤ 12.82 cm^3^ and N_0–1_	9 (42.86)	12 (57.14)		
Volume ≤ 12.82 cm^3^ and N_2–3_	9 (17.65)	42 (82.35)		

**P < 0.01, ***P < 0.001

## Discussion

Hypothyroidism after radiotherapy for nasopharyngeal cancer is a common late complication. The incidence of HT was 78.4, 56.4, and 43.4% in patients with nasopharyngeal cancer after receiving radiotherapy for 1, 2, and 3 years, respectively [10], which is similar to our results. In the present study, there was 50.49% HT incidence after radiotherapy in patients with NPC, and it encompassed clinical and subclinical HT. It was obvious that the incidence of HT in patients with NPC was not controlled effectively despite all studies suggesting the correlation between thyroid dose–volume and HT. What we know so far is that the risk factors for radiation-induced HT are sex, age, T stage, N stage, chemotherapy, thyroid volume, and radiation dose [16–18]. However, the dosimetric parameter cut-offs related to HT remain unclear. In the present study, we not only analyzed the effect of dosimetric parameters on thyroid function, but also combined the clinical factors and thyroid dosimetric parameters to predict the incidence of HT.

### Hypothyroidism and N stage

Wu et al. [17] found that N stage did not affect the incidence of HT, which is not consistent with our results. Our results show that patients with advanced N stage (N_2_ and N_3_) were at greater risk of developing HT. Here, the incidence rate of HT in the N_0–1_ and N_2–3_ groups was 13.11 and 37.38%, respectively. Logistic regression analysis showed that patients with N_2–3_ disease had 0.91 times increased risk of HT compared to patients with N_0–1_ disease. It might be because the thyroid gland in patients with cervical lymph node metastasis inevitably receives more radiation.

### Hypothyroidism and thyroid volume

Thyroid volume is closely related to the incidence of HT [19, 20], and we found a clear relationship between thyroid volume and radiation-induced HT (Table 3). The incidence of HT was 37.31% in patients with thyroid volume ≥ 12.82 cm^3^, while it was 75.% in patients with thyroid volume < 12.82 cm^3^. Therefore, we postulated that when the thyroid volume is small, there may be insufficient functional thyroid submits to produce thyroid hormone. Therefore, TSH increases as a normal feedback mechanism from the pituitary gland, and these patients are prone to developing radiation-induced HT [10].

### Hypothyroidism and thyroid dose

Currently, scholars here and abroad hold different opinions on the relationship between thyroid dose–volume and HT. One study suggested that the thyroid minimum dose, mean dose, and V_25_–V_60_ are significantly associated with HT [21]. Sommat et al. [22] have suggested that the thyroid V_40_ is closely related to HT, where the incidence of radiation-induced HT in the thyroid V_40_ ≤ 85% and thyroid V_40_ > 85% groups was 21.4% (3/14) and 61.4% (54/88), respectively. However, with our large sample, we show that HT incidence in the thyroid V_40_ ≤ 85% group was 47.93% (81/169), which is not consistent with the earlier study. This difference may be related to the follow-up time, sample size, and treatment planning systems. Thyroid V_50_ is an important prognostic variable for radiation-induced HT in patients with NPC [23]. For example, Ling et al. [24] retrospectively analyzed 102 patients and recommended thyroid V_50_ < 50% as the optimal dose–volume limiting threshold for HT after radiotherapy. Sachdev et al. [25] believed that thyroid V_50_ > 60% was a risk factor associated with the incidence of HT. Zhai et al. [26] found that patients with thyroid mean dose ≥45 Gy had 4.9 times increased risk of HT than those receiving a lower mean dose, and the recommended plan optimization objectives were reducing the thyroid V_45_ and V_50_ to 50 and 35%, respectively. Their results are similar to ours in that the thyroid mean dose, V_20_, and V_50_ were significantly correlated with HT, and thyroid V_50_ ≤ 24% was an independent predictor of HT. The incidence of HT was 34.15% in patients with thyroid V_50_ ≤ 24% and was 54.55% in patients with thyroid V_50_ > 24%.

### Hypothyroidism and combined factors

Based on the above results, more attention should paid to patients with small thyroid volume and advanced N stage. Therefore, we built combined factor models to guide individualized treatment. For each component model, thyroid volume and thyroid V_50_ (*P* < 0.001), thyroid volume and N stage (*P* < 0.001), and N stage and thyroid V_50_ (*P* < 0.01) were significantly different between patients with and without HT. Most previous studies focused only on thyroid dose, and neglected other clinical factors. Ours is the first retrospective study to consider both dosimetric variables and clinical factors such as N stage to predict the incidence of radiation-induced HT, which could guide physicians in forming individualized radiotherapy plans according to clinical factors and help reduce the HT occurrence rate in high-risk patients with NPC receiving IMRT.

### Hypothyroidism and pituitary dose

The pituitary gland is the most important and complex endocrine gland. It secretes a variety of hormones, such as growth hormone, thyroid hormone, adrenocorticotrophic hormone, gonadotropin, and oxytocin. The pituitary gland is located in the sella turcica, which is just superior to the nasopharynx. It might receive a high dose during radiotherapy for NPC. Many researchers believe that patients with NPC have a higher risk of developing HT after radiotherapy than patients with other head and neck cancers, as the morbidity of HT is closely related to the pituitary function through the HPT axis. Lin [27] demonstrated that patients with NPC with high thyroid and pituitary doses (> 50 Gy) had the highest risk of thyroid abnormalities (83.3%), followed by patients with high thyroid dose and low pituitary dose (50%). Huang [18] reported that TSH and FT_4_ were correlated with the pituitary volume receiving doses of > 55 Gy. However, a recent study showed that the Dmean, V_30_, V_40_, V_50_ and V_55_ of the pituitary gland indicated no significant differences between the euthyroid and hypothyroid groups (*P* > 0.05) [28]. Similarly, we did not find a correlation for pituitary dose and radiation-induced damage to thyroid function. This may be because patients with secondary HT (those with low TSH and low FT_4_) related to pituitary-hypothalamus damage were not included in the study.

### Hypothyroidism and other factors

In addition to thyroid volume, N stage, and thyroid V_20_ and V_50_, other factors such as sex, chemotherapy, age, and T stage were also considered HT-related. It remains controversial whether sex worsens the adverse effect of radiotherapy-induced HT; many researchers believe that female patients have a higher risk of HT .[19, 24, 29] Fan et al. [16] observed 2.03 times increased HT incidence in women relative to men among 14,893 patients with NPC and 16,105 patients with other head and neck cancers. Yet, others disagree: Bhandare et al. [30] reported that HT incidence in female patients was no higher than that in male patients. Our data support the former conclusion.

Zhai [26] reported that younger age (< 49 years) was a significant predictor for clinical HT after radiotherapy. Wu et al. [17] suggested that subclinical HT is strongly correlated with female patients or patients with lower T stage (T_1–2_ vs. T_3–4_). However, Luo et al. [29] identified chemotherapy as one of the most predictive factors for radiation-induced HT. In our research, we found that chemotherapy and T stage had no obvious statistically significant relation to the incidence of HT. This could have been caused by the small number of patients who did not receive chemotherapy and who had early T stage (T_1–2_) disease.

Our results demonstrate that sex, N stage, thyroid volume, thyroid mean dose, and thyroid V_20_ and V_50_ were significantly different between patients with and without HT. Logistic regression analysis showed that N stage, thyroid volume, and thyroid V_50_ were independent predictors of HT. The strengths of our study are the large sample size as compared to other studies, the comparison with the baseline TSH level before and after treatment, the thyroid and pituitary gland counting by one physician, and being the first retrospective study to combine dosimetric variables and clinical factors. There are also some limitations to our findings. First, our patients lacked long-term follow-up (2 years). Second, all patients did not undergo thyroid function testing at the same time. Prospective studies should be conducted in the future to analyze the optimal limiting dose of the pituitary gland to further reduce the incidence of radiation-induced HT.

## Conclusion

The incidence of HT in patients with NPC after IMRT was significantly associated with N stage, thyroid volume, and thyroid V_50_. Patients who received radiotherapy for the cervical lymph nodes were at greater risk of developing HT. However, given the limitation of the retrospective nature of our study, further prospective studies are needed to verify our conclusion. More attention should be paid to patients with thyroid volumes of ≤12.82 cm^3^ and with advanced N stage disease. Additionally, the pituitary dosimetric parameters did not show a statistically significant correlation with thyroid function.

## Data Availability

The datasets supporting the conclusions of this article are included within the article.

## Abbreviations

AUC: Area under the curve
CI: Confidence interval
CT: Computed tomography
CT-sim: CT simulation
D1cc: The dose delivered volume of 1 cm^3^
DVH: Dose-volume histogram
FT_3_: Free triiodothyronine
FT_4_: Free thyroxine
GTV: Gross tumor volume
HPT: Hypothalamic-pituitary-thyroid
HT: Hypothyroidism
IMRT: Intensity-modulated radiotherapy
MRI: Magnetic resonance imaging
NPC: Nasopharyngeal carcinoma
OARs: Organs at risk
OR: Odds ratio
PTV: Planning target volume
QoL: Quality of life
ROC: Receiver operating characteristic
RTOG: Radiation Therapy Oncology Group
TSH: Thyroid stimulating hormone
